# Evaluation of the Microcirculation in Chronic Thromboembolic Pulmonary Hypertension Patients: The Impact of Pulmonary Arterial Remodeling on Postoperative and Follow-Up Pulmonary Arterial Pressure and Vascular Resistance

**DOI:** 10.1371/journal.pone.0133167

**Published:** 2015-08-07

**Authors:** Takayuki Jujo, Seiichiro Sakao, Hatsue Ishibashi-Ueda, Keiichi Ishida, Akira Naito, Toshihiko Sugiura, Ayako Shigeta, Nobuhiro Tanabe, Masahisa Masuda, Koichiro Tatsumi

**Affiliations:** 1 Department of Respirology (B2), Graduate School of Medicine, Chiba University, 1-8-1, Inohana, Chuo-Ku, Chiba, 260–8670, Japan; 2 Department of Advanced Medicine in Pulmonary Hypertension, Graduate School of Medicine, Chiba University, 1-8-1 Inohana, Chuo-Ku, Chiba, 260–8670, Japan; 3 Department of Pathology, National Cerebral and Cardiovascular Center, 5-7-1, Fujishiro-Dai, Suita City, Osaka, 565–8565, Japan; 4 Department of Cardiovascular Surgery, Graduate School of Medicine, Chiba University, 1-8-1 Inohana, Chuo-Ku, Chiba, 260–8670, Japan; 5 Department of Cardiovascular Surgery, Chiba Medical Center, National Hospital Organization, 4-1-2, Tsubakimori, Chuo-ku, Chiba, 260–8606, Japan; VU University Medical Center, NETHERLANDS

## Abstract

**Background:**

Chronic thromboembolic pulmonary hypertension (CTEPH) is generally recognized to be caused by persistent organized thrombi that occlude the pulmonary arteries. The aim of this study was to investigate the characteristics of small vessel remodeling and its impact on the hemodynamics in CTEPH patients.

**Methods and Results:**

Hemodynamic data were obtained from right heart catheterization in 17 CTEPH patients before pulmonary endarterectomy (PEA). Lung tissue specimens were obtained at the time of PEA. Pathological observations and evaluation of quantitative changes in pulmonary muscular arteries and veins were performed using light microscopy on 423 slides in 17 patients. The relationship between the results and the hemodynamics of CTEPH was investigated. Pulmonary arteriopathy and venopathy were recognized in most cases, although no plexiform lesions and no capillary-hemangiomatosis-like lesions were detected in any of the specimens. The severity of pulmonary arteriopathy was correlated with pulmonary vascular resistance (PVR) in the postoperative and follow-up periods. The PVR and mean pulmonary arterial pressure were significantly higher in the high-obstruction group than in the low-obstruction group. The findings in pulmonary venopathy were similar to the findings seen in pulmonary veno-occlusive disease in some cases, although severe venopathy was only observed in a portion of the pulmonary veins. There was a significant correlation between the extent of pulmonary arteriopathy and venopathy, although an effect of pulmonary venopathy to hemodynamics, including pulmonary arterial wedged pressure (PAWP), could not be identified.

**Conclusion:**

The vascular remodeling of the pulmonary muscular arteries was closely associated with the hemodynamics of CTEPH. Severe pulmonary arteriopathy might be related to residual pulmonary hypertension after PEA. Those altered pulmonary arteries might be a new target for the persistent PH after the operation.

## Introduction

Chronic thromboembolic pulmonary hypertension (CTEPH) is generally characterized by the presence of persistent intravascular organized thrombi that partially or completely occlude the pulmonary arteries, leading to an elevated pulmonary arterial pressure (Ppa)[1–3]. Pulmonary endarterectomy (PEA), a surgical procedure used to remove organized thrombi up to the subsegmental pulmonary arteries, is the gold standard treatment for CTEPH [4,5]. However, about 10% of CTEPH patients achieve no hemodynamic improvements and continue to have persistent pulmonary hypertension (PH) despite successful PEA [4–6]. It has been suggested that obstruction of the proximal pulmonary arteries is not always associated with an elevated PVR in CTEPH patients [7,8]. Therefore, the distal pulmonary arteries may be involved in the pathogenesis of residual PH and re-exacerbation of PH after PEA in some patients with CTEPH [1,6]. Several histopathological studies have suggested that the features of pulmonary arteriopathy in the setting of CTEPH closely resemble those of pulmonary arterial hypertension (PAH) [9–12]. The vascular morphologic changes have recently been recognized in areas both with and without proximal thrombi in animal models [13] and clinical investigations [11,12].The small vessel remodeling distal to both obstructed and non-obstructed elastic pulmonary arteries has been demonstrated to play a potential role in the long-term progression of PH in patients with CTEPH, which is the so-called “small vessel disease hypothesis” [1,6]. However, there is little previous information on the relationship between pathological alterations in the pulmonary microvasculature and the hemodynamic data obtained from right heart catheterization (RHC) of CTEPH patients. Thus, the aim of this study was to investigate the characteristics of small vessel remodeling and its impact on hemodynamics in CTEPH patients.

## Methods

### Ethics statement

This study was approved by the Ethics Committee of Chiba University (approval number: 1221), and all patients provided their written informed consent for participation.

### Subjects

There were 17 patients diagnosed with CTEPH at Chiba University Hospital from October 2010 to September 2013 who were enrolled in this study. CTEPH was defined as elevation of the mean pulmonary pressure (mean Ppa ≥ 25 mmHg) with dyspnea persisting for more than six months, segmental defects on lung perfusion scans, and confirmed chronic pulmonary embolism on computed tomography (CT) or pulmonary angiography.

### Right heart catheterization (RHC)

A 7.5-Fr Swan-Ganz catheter (Edwards Lifesciences, USA) was used for RHC. The pulmonary arterial wedge pressure (PAWP) and the pressure in the right atrium, right ventricle, main pulmonary artery and right or left pulmonary artery were evaluated. The cardiac output (CO) was measured according to the thermodilution method. The cardiac index (CI) was subsequently calculated using the CO and body surface area, and the PVR was determined according to the following formula: PVR (dyne sec cm^-5^) = ((mean Ppa- PAWP) / CO) × 80. Hemodynamics were evaluated again by RHC at 1–2 months and approximately 12 months after PEA. We diagnosed a patient as persist PH after PEA when the postoperative PVR was higher than 500 dynes sec cm^-5^ [4].

### Indications for pulmonary endarterectomy

The indications for PEA were based on the following five criteria: 1) mean Ppa > 30 mmHg, 2) PVR > 300 dyne sec cm^-5^, 3) WHO functional class ≥ 2, 4) thrombi in the proximal pulmonary arteries, and 5) absence of significant comorbidities. In patients with mild PH (mean Ppa 25–30 mmHg), the decision to proceed with PEA depended on the willingness of the patient to undergo surgery based on an understanding of the risks of the procedure.

### Surgical procedure and biopsy

PEA was performed at Chiba Medical Center (Case 2) or Chiba University Hospital (Cases 1, 3–17) by the same two surgeons (Dr. K. I. and Dr. M. M.). A cubic centimeter of lung tissue (approximately, 1.0×0.5×0.5cm) was resected from the right middle lobe or left lingular segment during the PEA procedure for technical and safety reasons, and the number of specimens extracted from each patient was limited to only one for ethical and safety reasons. The selection of the areas for biopsy was left to the surgeon’s discretion. The specimens were perfused and fixed in 10% buffered formalin using a 24-gauge venous catheter at a pressure of 15–20 cmH_2_O. Following fixation, the specimens were embedded in paraffin, sliced into 3–4 μm serial sections and stained with Hematoxylin & Eosin or Elastica van Gieson stain. All slides were examined microscopically and recorded photographically using an Eclipse E400 (Nikon, Tokyo, Japan) and MOTICAM 1000 (Shimadzu Rika Corporation, Kyoto, Japan) system. All pathological findings were reviewed by both a trained pathologist and a pulmonologist.

### Connective tissue stain (modified Masson-Goldner stain)

The modified Masson-Goldner stain (Elastica Masson stain) was performed in order to know the characteristics of the pulmonary arteriopathy. The stain be useful in the differentiation of collagen (light-green), elatic fibers (dark purple), cells (red) [14]. The detail of the staining procedure was described in Text A in S1 File.

### Immunohistochemistry

Immunohistochemistry (IHC) was performed on deparaffinized glass slides using the automated immunostainer BOND-III (Leica, UK) according to the manufacturer’s protocol. We used mouse monoclonal antibodies as primary antibodies to stain for endothelial cells (CD34) (Novocastra, UK, dilution 1:200), for human α-smooth muscle actin (SMA) (DAKO, Japan, dilution 1:10), and for human D2-40 (DAKO, Japan, dilution 1:200) as a marker of lymphvessel endothelium. We also used polyclonal rabbit anti-human Von Willebrand Factor (DAKO, Japan, dilution 1:400). The detection kit as a secondary antibody was the Bond Polymer Refine Detection (Novocastra, DS9800), incubation with post primary for 8 minutes, polymer for 8 minutes, diaminobenzidine (DAB) for 10 minutes and Haematoxylin for 5 minutes.

### Evaluation of the pulmonary muscular arteries

For the quantification of the pulmonary arteriopathy and venopathy, sections were obtained at intervals of at least 20μm from each block and were stained with Elastica van Gieson stain. More than 20 sections from each patient were evaluated. There were a total of 423 slices prepared and evaluated in this study.

All pulmonary muscular arteries with adjacent bronchioles were examined and recorded, although the arteries were excluded when the horizontal-to-vertical ratio of these vessels was ≥1:2 or when the vascular diameter was ≥ 300μm. There were a total of 288 pulmonary muscular arteries quantified using the following methods. We adopted the "obstruction ratio”, which was modified from the “medial muscle mass” as described by Moser, et al [11]. The luminal and vascular areas were traced and measured using Image J software (ver. 1.45). The obstruction ratio is the ratio of the luminal and medial area to the vascular area of pulmonary muscular arteries (Fig A in S1 File), although “medial muscle mass” was the quantification of only the media [11]. The obstruction ratio of each pulmonary artery was calculated according to the following formula: Obstruction ratio = (1-luminal area)/vascular area. The mean obstruction ratio of each subject was calculated by dividing the total obstruction ratio by the number of arteries evaluated. The patients were divided into a high-obstruction group and a low-obstruction group based on the median value of the mean obstruction ratio. We analyzed both the differences of the two groups and the interaction between the hemodynamic data and the period of assessment. “The index of small pulmonary arteries in CTEPH” (IOCTEPH) was calculated for each subject as the average of four categorical scores that were assigned to the pulmonary arteries. The details of the methods were used to determine this index were described in a previous report [15].

### Evaluation of the pulmonary veins

All pulmonary veins included in each slide were examined (total 2,264 veins). Pulmonary veins could be identified as vessels which ran into the interlobular septa [16,17]. The vessels with developed adventitia, which ran into the interlobular septa, were thought to be pulmonary venules or veins. Vessels with thick external elastic layers and with unclear internal elastic layers were defined as pulmonary veins.

To quantify changes of pulmonary veins, we used the following methods. All pulmonary veins were detected on each slide of patients and classified into five categories: Score 0 (almost normal) included veins with single-layered endothelial cells; Score 1 (slight) included veins with slight intimal and/or medial thickening; Score 2 (mild) included veins with mild intimal and/or medial thickening (< 50% obstruction); Score 3 (moderate) included veins with highly intimal thickening (> 50% obstruction) and/or muscularization; and Score 4 (severe) included veins that were nearly obstructed (> 80% obstruction). “The mean PV score” for obtained by summing all scores and dividing by the numbers of the veins. The “%PV score of 3–4” in each case was defined as the frequency of the veins with a Score 3–4 to all of the veins.

### Statistical analysis

All data were analyzed using “EZR on R commander” statistical software (ver.1.27, http://www.jichi.ac.jp/saitama-sct/SaitamaHP.files/statmedEN.html)[18]. Continuous variables are described as the mean ± SD, and comparisons between two groups were made with the Mann-Whitney U-test, unless otherwise indicated. Time-dependent changes in the hemodynamic variables were analyzed using the Wilcoxon signed-rank test, and correlations between variables were assessed with Spearman's rank correlation coefficient. Multiple comparisons between a high-obstruction group and a low-obstruction group were analyzed by univariate repeated-measures ANOVA. The factors that were correlated with PVR by simple linear regression analyses were followed by stepwise regression analysis. A p-value of < 0.05 was considered significant.

## Results

### Patient characteristics

The characteristics of the study subjects are shown in Table 1. The age at diagnosis was 63.5 ± 9.1 years (range: 46–74). The disease duration from symptom onset to surgery was 40.6 ± 41.9 months (median: 22 months). Fifteen patients had a history of deep vein thrombosis. The preoperative hemodynamic findings among the 17 patients were as follows: mean Ppa was 44.6 ± 11.2 mmHg; PVR was 725 ± 307 dynes sec cm^-5^; CI was 2.95 ± 0.82 L/min/m^2^; and PAWP was 8.2 ± 2.8 mmHg.

**Table 1:** Clinical characteristics of the subjects.

	All cases (n = 17)	Male (n = 5)	Female (n = 12)	Gender difference
Age (years)	63.5 ± 9.1	59.0 ± 8.7	64.9 ± 9.1	N.S
Disease duration (months)	40.6 ± 41.9	23.8 ± 13.5	45.8 ± 46.6	N.S
A history of deep vein thrombosis	15	3	12	N.S
**Preoperative hemodynamics (n = 17)**				
Mean Ppa (mmHg)	44.6 ± 11.2	39.3 ± 9.0	46.2 ± 11.6	N.S
PVR (dyne sec cm^-5^)	725 ± 307	765 ± 356	713 ± 305	N.S
CI (L/min/kg)	2.95 ± 0.82	2.23 ± 0.72	3.17 ± 0.73	0.04*
PAWP (mmHg)	8.2 ± 2.8	8.0 ± 3.2	8.3 ± 2.8	N.S
**Postoperative hemodynamics (n = 16)**				
Mean Ppa (mmHg)	26.3 ± 10.1	24.3 ± 12.9	26.8 ± 9.9	N.S
PVR (dyne sec cm^-5^)	319 ± 170	278 ± 229	329.0 ± 164	N.S
CI (L/min/kg)	3.24 ± 0.47	3.03 ± 0.37	3.29 ± 0.49	N.S
PAWP (mmHg)	8.1 ± 2.7	7.7 ± 2.5	8.2 ± 2.9	N.S
Delta PVR (dyne sec cm^-5^)	434 ± 279	652 ± 250	384 ± 269	N.S
**Follow-up hemodynamics (n = 16)**				
Period after surgery (months)	12.6 ± 1.7	13.9 ± 2.2	12.4 ± 1.7	N.S
Mean Ppa (mmHg)	27.7 ± 9.2	26.7 ± 9.0	27.9 ± 9.6	N.S
PVR (dyne sec cm^-5^)	364 ± 193	339 ± 192	370 ± 200	N.S
CI (L/min/kg)	2.88 ± 0.70	2.39 ± 0.23	3.00 ± 0.72	N.S
PAWP (mmHg)	8.8 ± 2.5	9.3 ± 1.5	8.6 ± 2.7	N.S
**Pulmonary arteriopathy and venopathy (n = 17)**				
Obstruction ratio	0.824 ± 0.135	0.768 ± 0.099	0.841 ± 0.143	N.S
PV score	1.3 ± 0.4	1.0 ± 0.2	1.4 ± 0.4	N.S

*: p<0.05, analyzed according to the Mann-Whitney U-test

Ppa: pulmonary arterial pressure; PVR: pulmonary vascular resistance; CI: cardiac index; PAWP:pulmonary arterial wedge pressure; N.S: not significant.

The postoperative data (n = 16) obtained 1–2 months after surgery were as follows: mean Ppa was 26.3 ± 10.1 mmHg; PVR was 319 ± 170 dynes sec cm^-5^; CI was 3.24 ± 0.47 L/min/m^2^; and PAWP was 8.1 ± 2.7 mmHg. The delta PVR, the difference between the pre- and postoperative values, was 434 ± 279 dynes sec cm^-5^. The mean postoperative Ppa and PVR were significantly less than the preoperative values (mean Ppa: p = 0.0005, PVR: p = 0.00006).

Follow-up RHC studies (n = 16) were performed at 12.6 ± 1.7 months after PEA and the following hemodynamic data were obtained: mean Ppa was 27.7 ± 9.2 mmHg; PVR was 364 ± 193 dynes sec cm^-5^; and CI was 2.88 ± 0.70 L/min/m^2^; and PAWP was 8.8 ± 2.5 mmHg. Mean Ppa and PVR at follow-up were significantly lower than the preoperative values (mean Ppa: p = 0.0005, PVR: p = 0.00006). The follow-up PVR was significantly higher than the postoperative PVR, whereas there was no significant difference between the postoperative and follow-up mean Ppa (mean Ppa: p = 0.5, PVR: p = 0.04). No gender differences in the data were observed except for preoperative CI. Only one patient (Case 10) died after PEA, and this occurred 15 days after the procedure due to sudden respiratory failure of unknown cause. No complications associated with the lung biopsies were documented (including Case 10).

### An overview of pathological findings of the pulmonary microcirculation from pulmonary arteriopathy to venopathy

**Fig 1:**
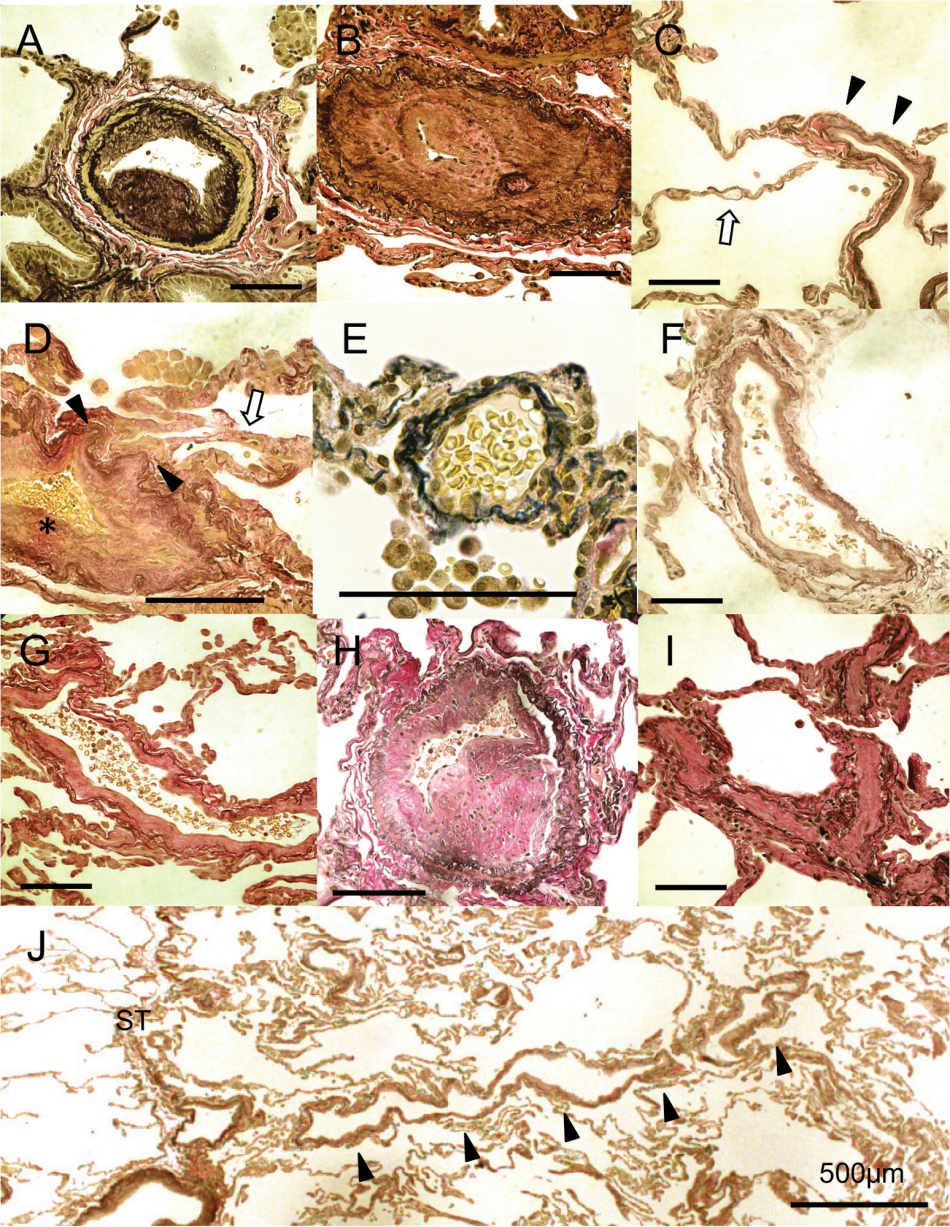
Representative photographs of the pulmonary arterioles, the pre- and post capillary vessels and the pulmonary venules. (A) Fibrous intimal thickening and mild medial thickening of the pulmonary muscular arteries (Case 2). (B) Cellular intimal thickening of the pulmonary muscular arteries resulting in obstruction of the lumen (Case 4). (C) Remodeling of post capillary vessels (arrow heads) (Case 10). The structures of capillaries did not show hemangiomatous appearance (arrow). (D) A remodeling lesion in the postcapillary vessels (arrow heads). The capillaries collapsed due to the obstruction of the proximal vessels (skeleton arrow) (Case 7) (*: Initimal fibrous thickening of a pulmonary vein is shown). (E) A venule next to the interlobular septa without intimal thickening (PV score 0) (Case 7). (F) A pulmonary vein with slight intimal thickening next to the interlobular septa (PV score 1) (Case 10). (G) A pulmonary venule with fibrotic intimal thickening (PV score 2) (Case 9). (H) A muscularized pulmonary vein with marked intimal thickening and moderate medial alterations. It is similar to the structure of pulmonary muscular arteries (Score 3) (Case 1). (I) Pulmonary venules with severe intimal thickening resulting in luminal obstruction similar to the pathology of pulmonary veno-occlusive disease (PVOD) (PV score 4) (Case 4). (J) The remodeling seems to extend from the post capillary vessels to the interlobular septum (Case 10). (All slides were stained with Elastica van Gieson stain. Scale bars shows 100 μm unless otherwise stated)

**Fig 2:**
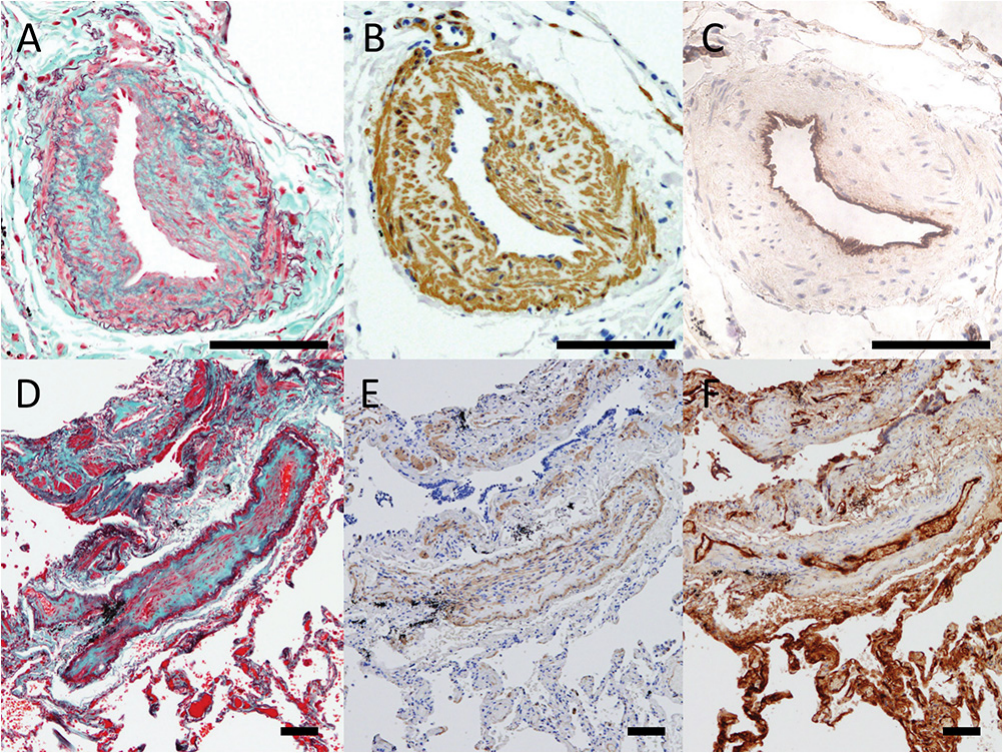
Modified Masson-Goldner stain and immunohistochemistry of pulmonary arteriopathy and venopathy. A to C show pulmonary muscular arteries from Case 17 with intimal fibrotic changes and moderate medial thickening. (A) Modified Masson-Goldner stain (Elastica Masson stain) shows the complicated arrangement of collagen (green), elastin (dark-purple) and spindle cells (red) within the intima. (B) Numerous α-smooth muscle actin (SMA) positive cells are recognized in the thickened intima and media in the artery. (C) Factor VIII is negative within the intima and media. Only endothelial cells are positive for factor VIII. D to F show pulmonary venules from Case 10 with intimal alterations. (D) Modified Masson-Goldner stain show that the neointima of venopathy is rich in collagen (green). (E) α-SMA-positive cells can be seen in the media and thin intima; however, the number of the cells is less than that seen in pulmonary arteriopathy. (F) Factor VIII is only positive in the endothelial cells. (Scale bars show 100 μm)

Medial hypertrophy and intimal thickening of the pulmonary muscular arteries were recognized in most cases. Both types of intimal thickening with fibrous hyperplasia (Fig 1A) and cellular hyperplasia of the pulmonary arteries (Fig 1B) were identified in most patients. In several cases, the lumen of the pulmonary arteries was occluded by severe intimal thickening (Fig 1B). No plexiform lesions, a hallmark of PAH, were recognized in the 423 slides in our study. Hemangiomatous structures were not found (Fig 1C) despite the sclerosis of precapillary and post capillary vessels although some capillaries were collapsed (Fig 1D). Capillary hemangiomatosis-like lesions were not recognized in any specimen. A wide range of pulmonary venopathy was seen the resected lung tissues (Fig 1E–1J, Table 2). Fibrotic intimal thickening of pulmonary veins was observed from the post capillary area to the interlobular septa (Fig 2G). Muscularized pulmonary veins with extensive intimal thickening and moderate medial alterations resembled the structure of pulmonary muscular arteries (Fig 2H). In five cases, severe and occlusive sclerotic lesions were detected in the pulmonary venules, similar to those documented in patients with pulmonary veno-occlusive disease (PVOD) (Fig 2I).The sclerotic changes were continuous from the post capillary area to the interlobular septa (Fig 2J).

**Table 2:** Summary of the pathological findings in the pulmonary vessels.

		Postoperative hemodynamics		Pulmonary arteries		Pulmonary veins					
Case	Biopsied lung side	Mean Ppa (mmHg)	PVR (dyne sec cm^-5^)	Mean obstruction ratio	Intimal thickening	Mean PV score	Score 0 (%)	Score 1 (%)	Score 2 (%)	Score 3 (%)	Score 4 (%)
**1**	Left	39	543	0.916	cellular	0.97	33.3	40.2	22.7	3.8	0
**2**	Left	15	139	0.718	fibrous	1.05	23.5	47.7	28.9	0	0
**3**	Left	15	115	0.592	(almost normal)	0.61	43.4	49.4	4.52	2.6	0
**4**	Left	48	570	0.962	cellular	1.51	12.6	46.2	27.3	5.6	8.4
**5**	Left	23	206	0.780	fibrous	0.89	21.1	68.7	10.2	0	0
**6**	Left	40	416	0.951	fibrous	1.86	4.3	31.4	48.6	5.7	10.0
**7**	Left	22	326	0.985	fibrous	1.61	13.2	36.8	31.6	12.6	5.8
**8**	Right	19	153	0.707	cellular	0.75	39.4	46.7	13.9	0	0
**9**	Right	16	128	0.724	fibrous	1.92	2.1	24.5	53.8	18.9	0.7
**10**	Right	No data	No data	0.730	fibrous	1.33	7.4	51.9	40.7	0	0
**11**	Left	33	375	0.977	fibrous	1.56	2.8	41.7	52.8	2.8	0
**12**	Left	18	88	0.552	fibrous	0.86	22.2	71.4	5.6	0	0.8
**13**	Left	19	255	0.863	cellular	1.10	14.9	61.9	21.6	1.5	0
**14**	Left	26	501	0.956	cellular	1.64	4.3	47.1	28.6	20.0	0
**15**	Left	23	357	0.861	cellular	1.39	7.8	47.1	43.2	1.9	0
**16**	Left	35	573	0.870	cellular	1.28	4.4	67.6	23.5	4.4	0
**17**	Left	30	368	0.864	cellular	1.34	16.0	48.0	22.0	14.0	0
**Mean**		**26.3 ± 10.1**	**319 ± 170**	**0.824±0.135**		**1.3±0.4**	**16.0±12.9**	**48.7±12.8**	**28.2±15.4**	**5.5±6.7**	**1.5±3.2**

### Connective tissue stain and immunohistochemistry of pulmonary arteriopathy and venopathy

Modified Masson-Goldner stain shows the complicated arrangement of collagen, elastin and spindle cells within the intima of pulmonary arteriopathy (Fig 2A). Numerous α-SMA-positive cells were seen in the thickened intima and media in the pulmonary muscular arteries of the biopsied specimens (Fig 2B). Cellular components within the intima and media were negative for factor VIII and CD34, although the endothelial cells which lined the luminal area, were positive (Fig 2C). In pulmonary venules, the neointima of venopathy was rich in collagen (Fig 2D). α-SMA-positive and factor VIII-negative cells were observed in the intima (Fig 2E and 2F); however, the numbers of those cells were less than that in pulmonary arteriopathy. D2-40, as a marker of lymph vessels, was negative in both pulmonary vessels with both arteriopathy and venopathy (data not shown).

### The Pulmonary arteriopathy could impact on hemodynamic data

**Fig 3:**
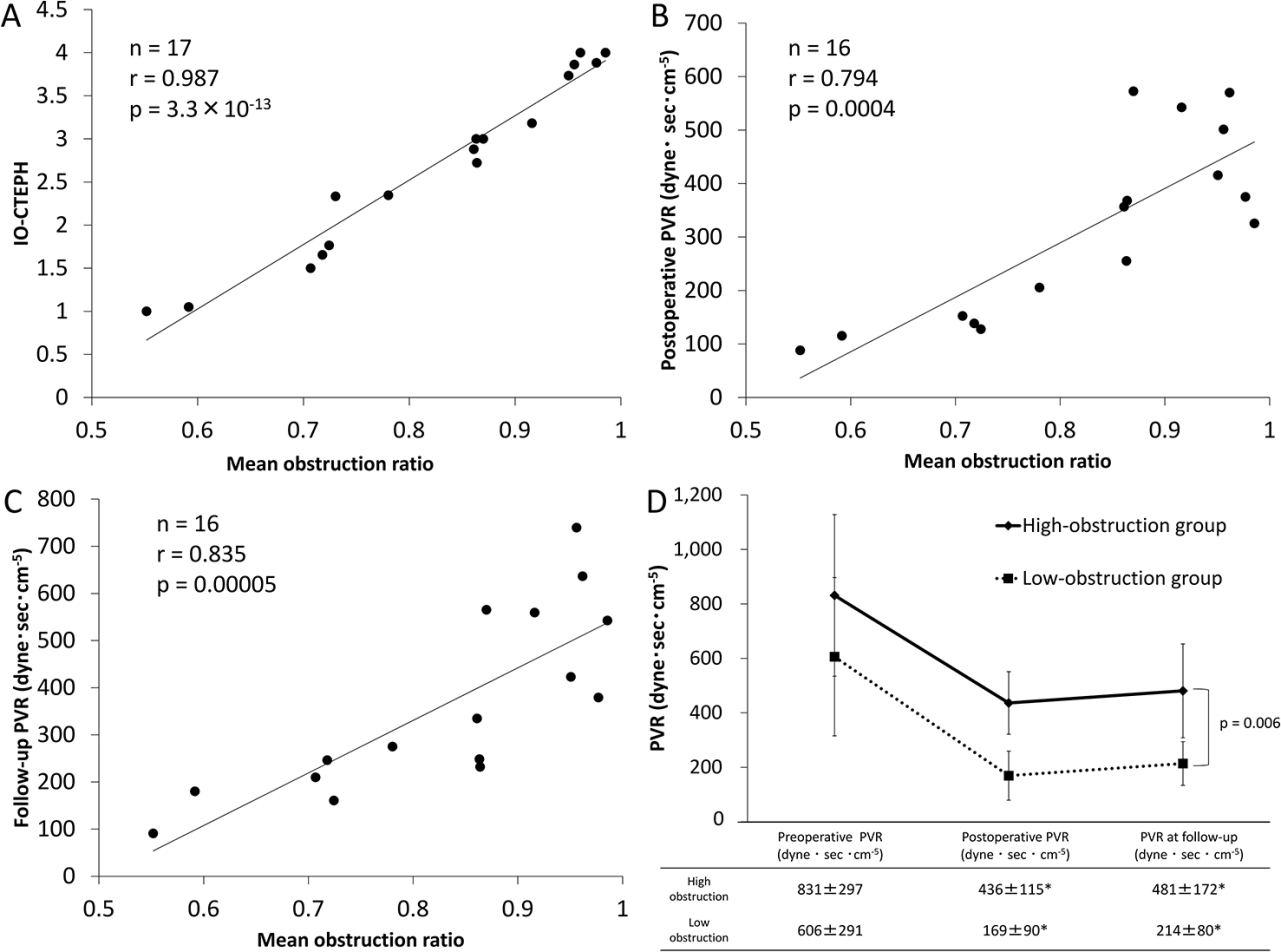
The impact of the pulmonary arteriopathy on the pulmonary vascular resistance (PVR). (A) The mean obstruction ratio is positively correlated with IOCTEPH which was another index of pulmonary arteriopathy (n = 17, r = 0.987, p = 3.0×10^−13^). (B) A significant positive correlation is observed between the postoperative PVR and the mean obstruction ratio (n = 16, r = 0.794, p = 0.0004). (C) The PVR obtained at the follow-up examination is correlated with the mean obstruction ratio (n = 16, r = 0.835, p = 0.00005) (these data were analyzed by Spearman's rank correlation). (D) The PVR is significantly greater in the high-obstruction group (mean obstruction ratio ≥ 0.863, n = 8) than in the low-obstruction group (mean obstruction ratio, < 0.863, n = 7) (p = 0.006, analyzed by univariate repeated-measures ANOVA, *: p < 0.05 vs. preoperative PVR;, analyzed by Bonferroni test following univariate repeated-measures ANOVA).

There were 288 pulmonary muscular arteries in 17 patients evaluated in this study. The mean diameter of the measured vessels was 169.7 ± 56.2 μm (range: 63–297 μm). The mean obstruction ratio and IOCTEPH were 0.824 ± 0.135 and 2.7 ± 1.0, respectively. No gender differences were found in these two parameters (obstruction ratio: p = 0.2, IOCTEPH: p = 0.2). The significant correlation between the mean obstruction ratio and IOCTEPH was identified (n = 17, r = 0.987, p = 3.0×10^−13^, Fig 3A). The postoperative and follow-up PVR values were correlated with the mean obstruction ratio (postoperative: n = 16, r = 0.794, p = 0.00004; follow-up: n = 16, r = 0.835, p = 0.00005; Fig 3B and 3C). In addition, the age of the patients was positively correlated with the ratio, whereas the disease duration was not (n = 17, age: r = 0.648, p = 0.005; disease duration: -0.075, p = 0.8; Table A in S1 File). In patients with persist PH (postoperative PVR higher than 500 dynes sec cm^-5^), the mean obstruction ratio was higher, although the difference was not significant (persist PH patients: n = 4, mean obstruction ratio = 0.926 ± 0.04; non-persist PH patients: n = 12, mean obstruction ratio = 0.789 ± 0.144; p = 0.10; Fig B in S1 File). The mean obstruction ratios for each Jamieson classification were as follows: type I (n = 7), 0.789 ± 0.129; type II (n = 7), 0.859 ±0.164; type III (n = 3), 0.821 ± 0.079; and type IV (n = 0). No significant differences were observed in the mean obstruction ratio between the three groups (p = 0.7, analyzed by one-way ANOVA).

According to the median value of the mean obstruction ratio (0.863), differences in hemodynamics between the high-obstruction group (obstruction ratio ≥ 0.863, n = 8) and the low-obstruction group (obstruction ratio, < 0.863, n = 7) were investigated. Consequently, PVR and mean Ppa values were significantly greater in the high-obstruction group than in the low-obstruction group (analyzed according to univariate repeated-measures ANOVA; PVR: p = 0.005; mean Ppa: p = 0.02; Fig 3D and Fig C in S1 File). However, the interaction between the hemodynamics and the assessment period were not significant (analyzed according to univariate repeated-measures ANOVA; PVR: p = 0.7; mean Ppa: p = 0.6; data not shown).

### The luminal area of pulmonary muscular arteries and PVR

**Fig 4:**
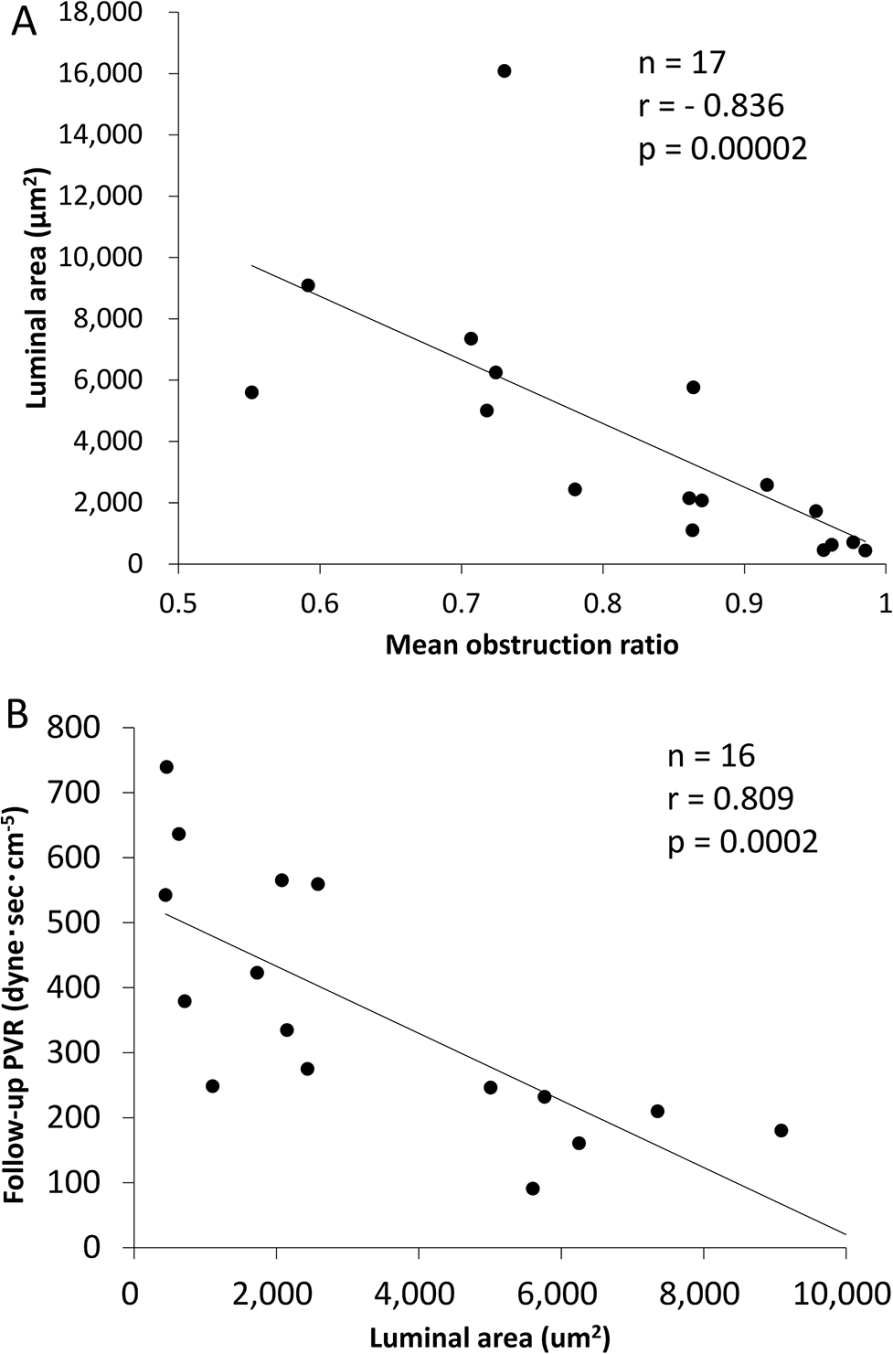
The correlations between luminal areas of the pulmonary arteries, the obstruction ratio and pulmonary vascular resistance. (A) The luminal areas of pulmonary arteries were negatively correlated with the mean obstruction ratio (n = 17, r = -0.836, p = 1.6×10^−5^). (B) The luminal area were negatively correlated with the follow-up pulmonary vascular resistance (PVR) (n = 16, r = -0.809, p = 0.0002).

The luminal and vascular areas also were obtained during calculation of the obstruction ratio. The mean luminal area was negatively correlated with the mean obstruction ratio (n = 17, r = -0.836, p = 0.00002, Fig 4A). The mean luminal area was also negatively correlated with the postoperative and follow-up PVR (Fig 4B and Table B in S1 File). These correlations were not observed in the analysis used mean vascular area (Table B in S1 File).

### Pulmonary venopathy and hemodynamic data

There were 2,264 veins from 17 cases investigated in this study. The mean percentages of each score in the 17 cases were as follows: Score 0, 1.3 ± 0.4%; Score 1, 16.0 ± 12.9%; Score 2, 48.7 ± 15.4%; Score 3, 5.5 ± 6.7%; and Score 4, 1.5 ± 3.2% (Table 2). The mean PV score was 1.3 ± 0.4 (Table 2). Gender difference was not found (p = 0.1). The PV score was not correlated with the clinical data and hemodynamics except for the follow-up mean Ppa (r = 0.516, p = 0.04, Table C in S1 File). It was noteworthy that the mean PV score was not correlated with PAWP during any of the three time periods (preoperative PAWP: r = 0.0313, p = 0.9; postoperative PAWP: r = -0.0313, p = 0.6; follow-up PAWP: r = -0.184, p = 0.5; Table C in S1 File).

### The relationship between pulmonary arteriopathy and venopathy

**Fig 5:**
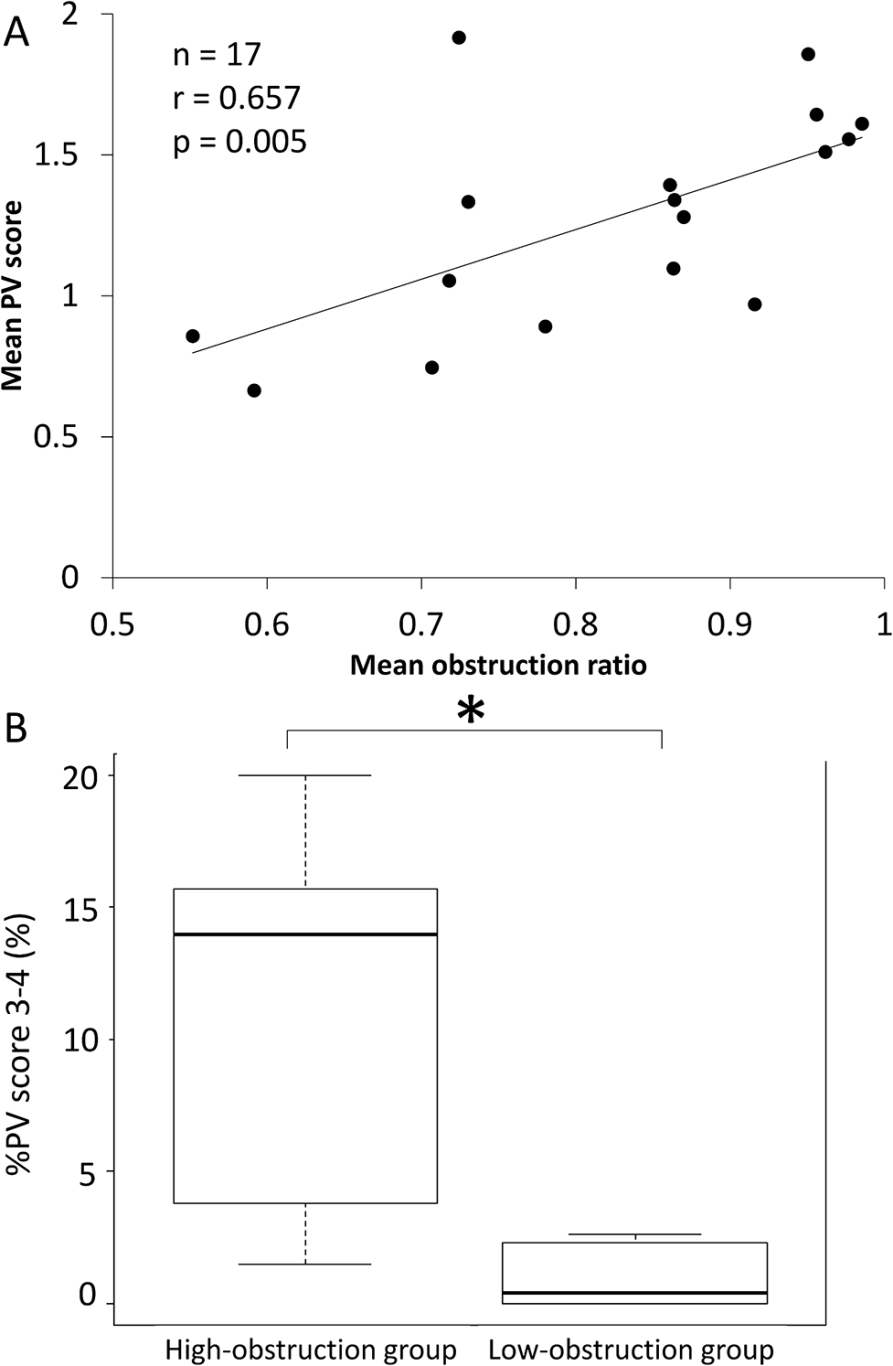
The relationship between the extent of remodeling of the pulmonary arteries and veins. (A) The mean PV score had a significant positive correlation with the mean obstruction ratio (n = 17, r = 0.657, p = 0.005). (B) In the high-obstruction group, the % PV score 3–4 (the fraction of all pulmonary veins with moderate-to-severe alternation) were significantly high compared with the ratio in the low-obstruction group (p = 0.01, analyzed by Wilcoxon’s signed-rank test).

There was a significant correlation between the mean obstruction ratio of the pulmonary arteries and the mean PV score (r = 0.657, p = 0.005, Fig 5A). The %PV score of 3–4 were significantly higher in the high-obstruction group than in low-obstruction group (p = 0.01, Fig 5B). Simple linear regression analyses suggested that the age of patients and the mean obstruction ratio might also be correlated with the postoperative and follow-up PVR. However, stepwise multiple regression analysis revealed that only the mean obstruction ratio was correlated with the postoperative and follow-up PVR (Tables 3 and 4).

**Table 3:** The results of univariate and multivariate analysis to determine the variables associated with postoperative pulmonary vascular resistance.

	Simple linear regression analysis		Multiple linear regression analysis	
	SRC	p-value	SRC	p-value
**Age**	0.52	0.02	-0.025	0.9
**Obstruction ratio**	0.81	0.0002	0.82	0.002
**PV score**	0.41	0.09		

SRC: standardized regression coefficient

**Table 4:** The results of univariate and multivariate analysis to determine the variables associated with follow-up pulmonary vascular resistance.

	Simple linear regression analysis		Multiple linear regression analysis	
	SRC	p-value	SRC	p-value
**Age**	0.57	0.02	0.086	0.7
**Obstruction ratio**	0.78	0.0002	0.72	0.007
**PV score**	0.42	0.09		

SRC: standardized regression coefficient

## Discussion

This study showed that the remodeling of pulmonary muscular arteries could impact post operative and follow-up hemodynamics in CTEPH patients. Sclerotic changes in pulmonary veins were associated with remodeling of pulmonary muscular arteries; however, these changes were not associated with hemodynamics.

The severity of pulmonary arteriopathy was closely associated with postoperative and follow-up hemodynamics obtained by RHC (Fig 3B and 3C, Tables 3 and 4 and Table A in S1 File). The mean obstruction ratio could be as appropriate an index of arteriopathy as IOCTEPH (Fig 2). The luminal area was correlated with not only the mean obstruction ratio but also the postoperative and follow-up PVR (Fig 3B, Table B in S1 File). Poiseuille’s formula (PVR = (8×L×η) / (π×r^4^) (L: length of vessels, η: blood viscosity, r: cross-sectional radius of vessels) [19] indicates that vessels with the narrower lumen have greater resistance. Our results suggested that the close relationship between severe pulmonary arterial remodeling and the elevated PVR could be due to luminal narrowing of pulmonary muscular arteries. Moser et al. reported that their quantification parameter, the “medial muscle mass,” exhibited no correlation with the preoperative mean Ppa and PVR values, although they had no postoperative or follow-up hemodynamic data [11]. Moreover, Moser et al did not provide precise diagnostic criteria for CTEPH [11]. This discrepancy may be related to differences in the diagnostic approach to detecting CTEPH based on our criteria.

Clinically, the residual pulmonary hypertension after PEA might be explained by the pulmonary arteriopathy. We found that the mean Ppa and PVR values were greater in the high-obstruction group than in the low-obstruction group at all time points (Fig 3D and Fig C in S1 File). It was also suggested that the obstruction of pulmonary arteries could be higher in patients with persist PH after PEA (Fig B in S1 File). In particular, the postoperative and follow-up PVR values were significantly higher in the patients with the most severe degree of vascular obstruction (obstruction ratio > 0.9) (Fig 3B and 3C). Previous reports have suggested that pulmonary arteriopathy induces vascular obstruction and/or stenosis, and this may play an important role in the elevation of PVR in CTEPH patients [1,2,19] as well as the persistence of PH and poor prognosis after PEA [2,15,20]. The altered pulmonary arteries might be a new target for treatment of persistent PH after the operation. It may well be useful to assess the pulmonary microvasculature in CTEPH patients; however, biopsy during the operation is too invasive to be performed routinely. The assessment of the degree of subpleural perfusion using pulmonary angiography [21] and the measurement of the occlusion pressure of the subsegmental pulmonary arteries [22,23] might be less invasive methods for predicting the prognosis after PEA. It is therefore necessary to develop a noninvasive method for estimating the degree of vasculopathy in the near future.

Pulmonary venopathy with musucularization or obstruction was recognized in several cases; however, the pathological alteration of 70–80% of pulmonary venules could remain slight or show mild changes even in the areas showing severe arteriopathy or venopathy (Table 2). This might be responsible for the poor correlation between the hemodynamic data including PAWP and the extent of pulmonary venopathy (Table C in S1 File). Recently, several reports have described sclerotic lesions in the pulmonary venules of patients with CTEPH [15,24], idiopathic PAH [25] and PAH associated with connective tissue disease [26]. It seemed that pulmonary venopathy was a common factor with several types of pulmonary hypertension. It was also reported that severe pulmonary venopathy was observed in the lung area where there was severe obstruction of the pulmonary arteries in CTEPH animal models [24] or lung biopsy specimens from CTEPH patients [15]. Those reports support our results (Fig 4A and 4B). Various causes of the sclerotic changes of pulmonary veins have been proposed [27]. The intimal thickening of the pulmonary veins was usually observed in older patients [16,17], although a correlation between the age of patients and mean PV score was not found in the current study (Table C in S1 File). Dorfmüller et al. reported that pulmonary venopathy might be affected by bronchopulmonary shunts [24], although the bronchopulmonary circulation was not investigated in our study. In this study, a relationship between the severity of pulmonary arteriopathy and venopathy was observed (Fig 5A and 5B). It is possible that the development and progression of pulmonary arteriopathy might be followed by secondary remodeling of the pulmonary veins. The precise mechanisms responsible for the development of pulmonary venopathy are still unclear based on our study, and further investigation will be needed.

In the operable CTEPH cases in this study, there were no plexiform lesions and no capillary hemangiomatosis-like lesion. Several studies reported that plexiform lesions were present in CTEPH lung specimens [10–12]. Dorfmüller et al. reported a capillary hemangiomatosis-like lesion recognized in lung tissues of CTEPH patients [24], although there was no proliferative lesion of the capillaries or capillary hemanigiomatosis-like lesion in this study. Lung tissues resected from operable CTEPH patients (not progressive phase) were investigated in both the current study and the study by Yamaki, et al. [15], whereas other investigations mainly assessed tissues removed from patients who required lung transplantation and/or died of CTEPH [10–12]. It is possible that the inconsistency of results among studies could be related to the severity of CTEPH.

α-SMA positive and Factor VIII negative cells were recognized within the thickened intima in pulmonary arteriopathy and venopathy (Fig 2). We speculated that the α-SMA positive and Factor VIII negative cells might be myofibroblast-like cells from the results of our previous study [28,29]. It was also reported that myofibroblast-like cells could migrate from the media to intima and proliferate within the intima [30].The crosstalk between the myofibroblast-like cells and the endothelial cells might be related to the pathobiological development of CTEPH [29] [31]. The significance of α-SMA-positive and Factor VIII-negative cells within areas of pulmonary venopathy was not apparent in this study. The information about component cells of pulmonary arteriopathy and venopathy was not adequate in this study, therefore further investigation was needed.

Our hypothesis regarding the relationship between the vascular remodeling and the hemodynamics in CTEPH can be explained as follows. The vascular remodeling has been present both in open and occluded vascular areas before PEA [11,12]. It is considered that endothelial dysfunction followed by growth factors released from proximal thrombi and abnormal cell proliferation can be related to developing distal vascular alternation in both areas [29,31,32]. Prior to PEA, open vascular beds without proximal thrombi are exposed to a high pulmonary arterial pressure, while the occluded areas are protected by proximal thrombi [33]. The PVR can be determined according to the degree of remodeling of the open vascular bed. After the organized thrombi are removed by PEA, the distal vascular bed of the occluded areas is exposed to a high pulmonary arterial pressure [33]. However, inhomogeneous perfusion is often observed after PEA as a vascular stealing phenomenon that improves during the follow-up period [34]. The presence of sclerotic pulmonary arteries in the occluded and non-occluded vascular areas may have an effect on hemodynamics, especially during the follow-up period. In fact, PEA can be used to remove the greater portion of the proximal thrombi and has been shown to provide hemodynamic improvements that are well-correlated with the amount of thrombus removed [35]. Conversely, the persistent PH after PEA could be due to the presence of residual proximal thrombi or distal vascular lesions [20,36]. The results of this study further suggest that the presence of distal vascular lesions could have an impact on hemodynamics in CTEPH patients, and those lesions might be new targets for the treatment of CTEPH.

Our study had some limitations. The sample size was small and only a single lung specimen was resected from each patient. Therefore, it is uncertain whether the pathological findings of the small vessel lesions evaluated indicate the presence of pathological changes in the entire lung vasculature resulting in disordered hemodynamics. Vasoconstriction of pulmonary arterioles using vasodilators or exercise stress was not evaluated in this study, and this is an important limitations. The time course of the changes in vascular remodeling after PEA is also unknown. Repeated examinations of multiple, larger lung specimens was desired, but could not be performed for ethical reasons [34].

## Conclusion

The current study demonstrated that vascular alterations of the pulmonary muscular arterioles could closely related the elevated mean Ppa and PVR after the PEA in operable CTEPH patients. Severe pulmonary arteriopathy might be related to residual pulmonary hypertension after PEA. Those altered pulmonary arteries might be a new target for the persistent PH after the operation.

## Supporting Information

S1 File Supporting information files. **Text A**, Staining procedure of modified Masson-Goldner stain. **Fig A**, The graphical interpretation of the obstruction ratio. **Table A**, The correlations between the obstruction ratio and the clinical values. **Fig B**, High obstruction of pulmonary arteries of patients with persist PH after PEA. **Fig C**, The mean pulmonary arterial pressure in high-obstruction and low-obstruction group. **Table B**, The correlations between the luminal and vascular areas and pulmonary vascular resistance. **Table C**, The correlations between the ‘mean PV score’ and the clinical values.
(PDF)
