# Correction: Evaluation of the Microcirculation in Chronic Thromboembolic Pulmonary Hypertension Patients: The Impact of Pulmonary Arterial Remodeling on Postoperative and Follow-Up Pulmonary Arterial Pressure and Vascular Resistance

**DOI:** 10.1371/journal.pone.0138040

**Published:** 2015-09-08

**Authors:** Takayuki Jujo, Seiichiro Sakao, Hatsue Ishibashi-Ueda, Keiichi Ishida, Akira Naito, Toshihiko Sugiura, Ayako Shigeta, Nobuhiro Tanabe, Masahisa Masuda, Koichiro Tatsumi

There is an error in Table 1. The values in the Male and Female columns are incorrect. The authors have provided a correct Table 1 here.

**Table 1:** Clinical characteristics of the subjects.

	All cases (n = 17)	Male (n = 4)	Female (n = 13)	Gender difference
Age (years)	63.5 ± 9.1	59.0 ± 8.7	64.9 ± 9.1	N.S
Disease duration (months)	40.6 ± 41.9	23.8 ± 13.5	45.8 ± 46.6	N.S
A history of deep vein thrombosis	15	3	12	N.S
**Preoperative hemodynamics (n = 17)**				
Mean Ppa (mmHg)	44.6 ± 11.2	39.3 ± 9.0	46.2 ± 11.6	N.S
PVR (dyne sec cm^-5^)	725 ± 307	765 ± 356	713 ± 305	N.S
CI (L/min/kg)	2.95 ± 0.82	2.23 ± 0.72	3.17 ± 0.73	0.04*
PAWP (mmHg)	8.2 ± 2.8	8.0 ± 3.2	8.3 ± 2.8	N.S
**Postoperative hemodynamics (n = 16)**				
Mean Ppa (mmHg)	26.3 ± 10.1	24.3 ± 12.9	26.8 ± 9.9	N.S
PVR (dyne sec cm^-5^)	319 ± 170	278 ± 229	329.0 ± 164	N.S
CI (L/min/kg)	3.24 ± 0.47	3.03 ± 0.37	3.29 ± 0.49	N.S
PAWP (mmHg)	8.1 ± 2.7	7.7 ± 2.5	8.2 ± 2.9	N.S
Delta PVR (dyne sec cm^-5^)	434 ± 279	652 ± 250	384 ± 269	N.S
**Follow-up hemodynamics (n = 16)**				
Period after surgery (months)	12.6 ± 1.7	13.9 ± 2.2	12.4 ± 1.7	N.S
Mean Ppa (mmHg)	27.7 ± 9.2	26.7 ± 9.0	27.9 ± 9.6	N.S
PVR (dyne sec cm^-5^)	364 ± 193	339 ± 192	370 ± 200	N.S
CI (L/min/kg)	2.88 ± 0.70	2.39 ± 0.23	3.00 ± 0.72	N.S
PAWP (mmHg)	8.8 ± 2.5	9.3 ± 1.5	8.6 ± 2.7	N.S
**Pulmonary arteriopathy and venopathy (n = 17)**				
Obstruction ratio	0.824 ± 0.135	0.768 ± 0.099	0.841 ± 0.143	N.S
PV score	1.3 ± 0.4	1.0 ± 0.2	1.4 ± 0.4	N.S

*: p<0.05, analyzed according to the Mann-Whitney U-test; Ppa: pulmonary arterial pressure; PVR: pulmonary vascular resistance; CI: cardiac index; PAWP:pulmonary arterial wedge pressure; N.S: not significant.
