# Supplementary material for: Evaluation of the Microcirculation in Chronic Thromboembolic Pulmonary Hypertension Patients: The Impact of Pulmonary Arterial Remodeling on Postoperative and Follow-Up Pulmonary Arterial Pressure and Vascular Resistance
Source: PLoS One. 2015 Aug 7;10(8):e0133167. doi: 10.1371/journal.pone.0133167 (PMC4529247; doi:10.1371/journal.pone.0133167)
Supplement: S1 File — Text A, Staining procedure of modified Masson-Goldner stain. Fig A, The graphical interpretation of the obstruction ratio. Table A, The correlations between the obstruction ratio and the clinical values. Fig B, High obstruction of pulmonary arteries of patients with persist PH after PEA. Fig C, The mean pulmonary arterial pressure in high-obstruction and low-obstruction group. Table B, The correlations between the luminal and vascular areas and pulmonary vascular resistance. Table C, The correlations between the ‘mean PV score’ and the clinical values. (PDF) [file pone.0133167.s001.pdf]

Supporting Information

Title

Evaluation of microcirculation in chronic thromboembolic pulmonary hypertension patients: Pulmonary arterial remodeling could impact on postoperative and follow-up pulmonary pressure and pulmonary vascular resistance

Authors

Takayuki Jujo, Seiichiro Sakao, Hatsue Ishibashi-Ueda, Akira Naito, Keiichi Ishida, Toshihiko Sugiura, Ayako Shigeta, Nobuhiro Tanabe, Masahisa Masuda, Koichiro Tatsumi.

Text A: Staining procedure of modified Masson-Goldner stain [1,2]

Preparation of staining solutions

3% potassium bichromate, resorcin-fuchsin fluid, Weigert's iron hematoxilin, Masson staining fluid B and light-green fluid were purchased from Muto Pure Chemicals Co,ltd, (Hongo, Bunkyo-Ku, Tokyo, Japan). Phosphomolybdic acid-orange G fluid: dissolve 3.5g phosphomolybdic acid and 2g orange G in 100ml distilled water.

Staining procedure

Procedures were unless otherwise state.

1. Deparaffinize and hydrate slides
2. Mordant sections in 3% potassium bichromate at 60 degrees Celcius for 60 minutes.
3. Wash sections in running water for 10 minutes
4. Stain in resorcin-fuchsin for 90 minutes.
5. Differentiate in ethanol containing 1% hydrochronic acid (HCl) for 30 seconds.
7. Wash sections in running water for 1 minute.
8. Weigert's iron hematoxilin stain for 5 minutes.
9. Wash sections in running water for 10 minutes.
10. Stain in Masson staining fluid B for 5 minutes.
11. Wash in 1% acetic acid for 5 seconds.
12. Stain in phosphomolybdic acid-orange G for 3 minutes.
13. Wash in 1% acetic acid for 5 seconds.
14. Place sections in 2.5% phosphotungstic acid for 7 minutes.
15. Wash sections in 1% acetic acid for 5 seconds.
16. Stain in light green fluid for 15 minutes.
17. Rince in 1% acetic acid.
18. Dehydrate, clear, and mount.

Reference of Text A

1. Watanabe M. Elastica-Masson Senshoku. Byouri-to-Rinshou 2010;28:334-338 (in Japanese).

2. Maruyama KK, K. Haga, H. Matsuno Y. Elastica-Masson's stain. In: Mizuguchi K, editor. Senshokuhou-no-Subete. Tokyo, Japan: Ishiyaku Publishers, Inc; 2011. pp. 16-18 (in Japanese).

Figure A: The graphical interpretation of the obstruction ratio. Vascular area and luminal area of each pulmonary artery were traced and measured using Image J software (ver. 1.45). The external elastic lamina was traced and the enclosed area was defined as the vascular area. The luminal wall was traced (blood cells were not included) and the enclosed area was defined as the luminal area. Obstruction ratio was the ratio of the intimal and medial area to vascular area. The obstruction ratio of each pulmonary artery was calculated according to the following formula: Obstruction ratio = (1-luminal area)/vascular area.

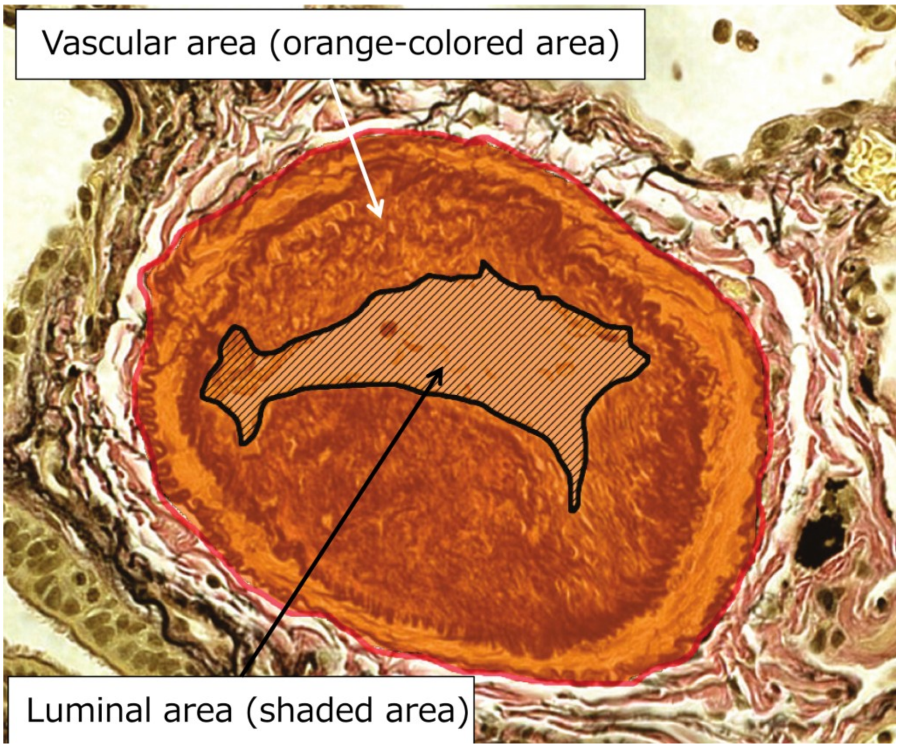

Table A: The correlations between the obstruction ratio and the clinical values

	rho	p-value
Age at diagnosis (years)	0.648	0.005
Disease duration (months)	-0.0748	N.S
Preoperative data		
Mean Ppa (mmHg)	0.515	0.03
PVR (dyne･sec･cm^{-5})	0.404	N.S
Cardiac index (L/min/m^{2})	-0.0441	N.S
PAWP (mmHg)	0.0225	N.S
Postoperative data		
Mean Ppa (mmHg)	0.743	0.001
PVR (dyne･sec･cm^{-5})	0.794	0.0004
Cardiac index (L/min/m^{2})	0.0118	N.S
PAWP (mmHg)	-0.158	N.S
Follow-up data		
Mean Ppa (mmHg)	0.783	0.0003
PVR (dyne･sec･cm^{-5})	0.835	0.00005
Cardiac index (L/min/m^{2})	0.0324	N.S
PAWP (mmHg)	-0.45	N.S

Ppa: pulmonary arterial pressure; PVR: pulmonary vascular resistance; CI: cardiac index. PAWP:pulmonary arterial wedged pressure, N.S: not significant

Figure B. High obstruction of pulmonary arteries of patients with persist PH after PEA. In patients with persist pulmonary hypertension (PH) (postoperative pulmonary vascular resistance (PVR) higher than 500 dynes・sec・cm^{-5}), the mean obstruction ratio was higher, although the difference was not significant (persist PH patients: n = 4, mean obstruction ratio = 0.926 ± 0.04; non-persist PH patients: n = 12, mean obstruction ratio = 0.789 ± 0.144; p=0.10, analyzed by Mann-Whitney U-test.).

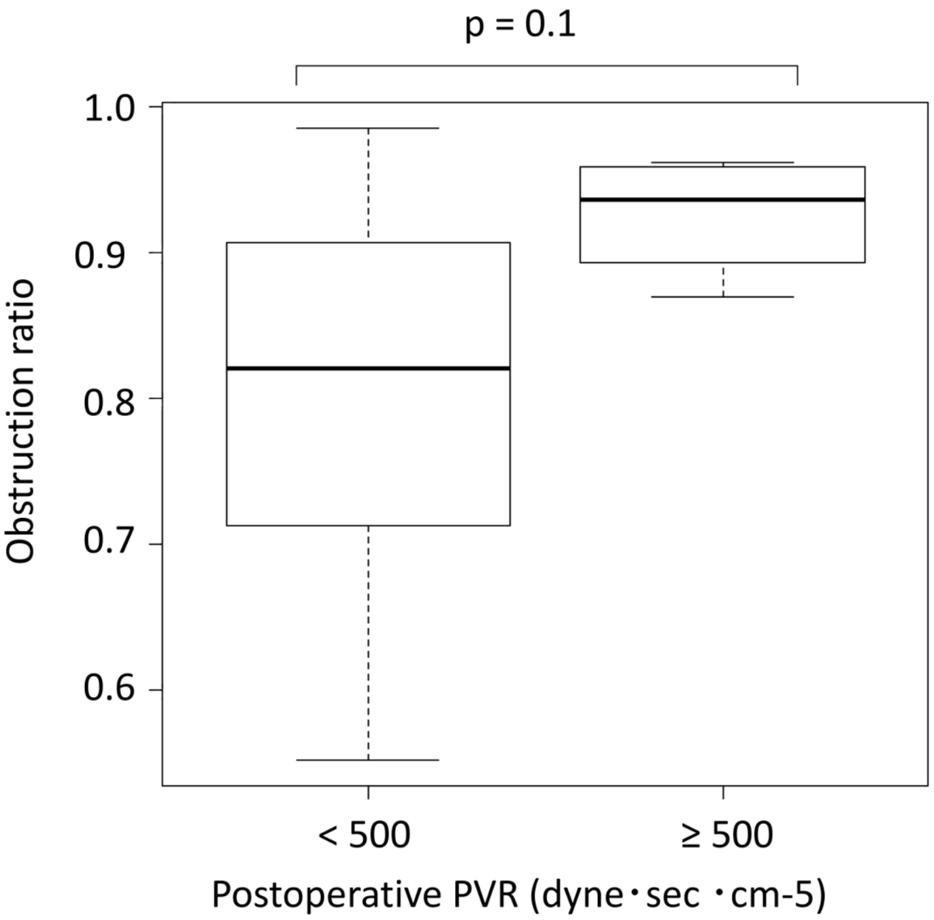

Figure C: The mean pulmonary arterial pressure in high-obstruction and low-obstruction group. The mean pulmonary arterial pressure (Ppa) is significantly greater in the high-obstruction group (mean obstruction ratio ≥ 0.863, n=8) than in the low-obstruction group (mean obstruction ratio, < 0.863, n=7) (p=0.004, analyzed by univariate repeated-measures ANOVA, *: p < 0.05 vs. preoperative mean Ppa, analyzed by Bonferroni test following univariate repeated-measures ANOVA).

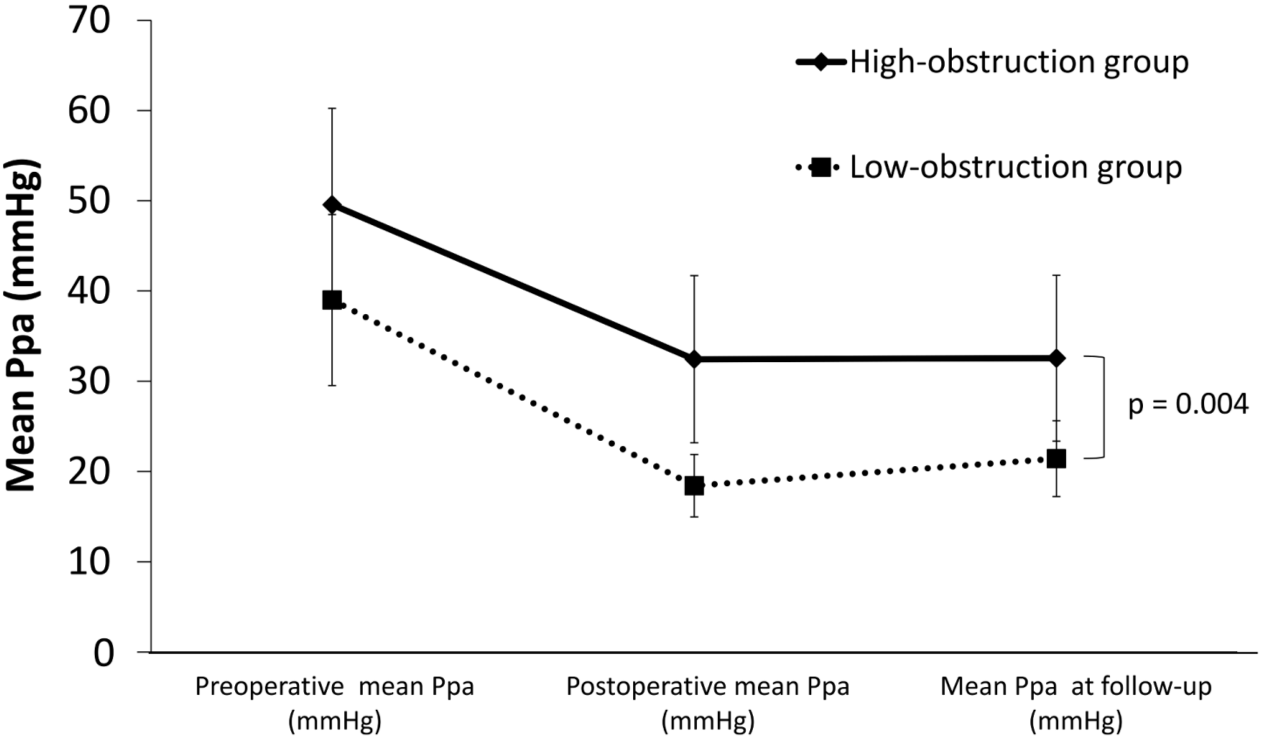

High obstruction	49.6±10.7	32.4±9.3*	32.6±9.2*
Mild obstruction	39.0±9.5	18.4±3.5*	21.4±4.2*

Table B: The correlations between the luminal and vascular areas and pulmonary vascular resistance

	Luminal area		Vascular area	
	rho	p-value	rho	p-value
Preoperative PVR	-0.311	N.S	0.324	N.S
Postoperative PVR	-0.626	0.0111	0.359	N.S
Follow-up PVR	-0.809	0.00022	0.2	N.S

PVR: pulmonary vascular resistance, N.S: not significant

Table C: The correlations between the 'mean PV score' and the clinical values.

	rho	p-value
Age at diagnosis (years)	0.292	N.S
Disease duration (months)	0.197	N.S
Prepoperative data		
Mean Ppa (mmHg)	0.472	N.S
PVR (dyne・sec・cm^{-5})	0.289	N.S
Cardiac index (L/min/m^2)	0.105	N.S
PAWP (mmHg)	0.0313	N.S
Postoperative data		
Mean Ppa (mmHg)	0.389	N.S
PVR (dyne・sec・cm^{-5})	0.426	N.S
Cardiac index (L/min/m^2)	0.00442	N.S
PAWP (mmHg)	-0.159	N.S
Follow-up data		
Mean Ppa (mmHg)	0.516	0.04
PVR (dyne・sec・cm^{-5})	0.438	N.S
Cardiac index (L/min/m^2)	0.249	N.S
PAWP (mmHg)	-0.184	N.S

Ppa: pulmonary arterial pressure; PVR: pulmonary vascular resistance; CI: cardiac index. PAWP:pulmonary arterial wedged pressure, N.S: not significa
